# Supplementary figures and images for: The response of geophytes to continuous human foraging on the Cape south coast, South Africa and its implications for early hunter-gatherer mobility patterns
Source: PeerJ. 2022 May 3;10:e13066. doi: 10.7717/peerj.13066 (PMC9074880; doi:10.7717/peerj.13066)

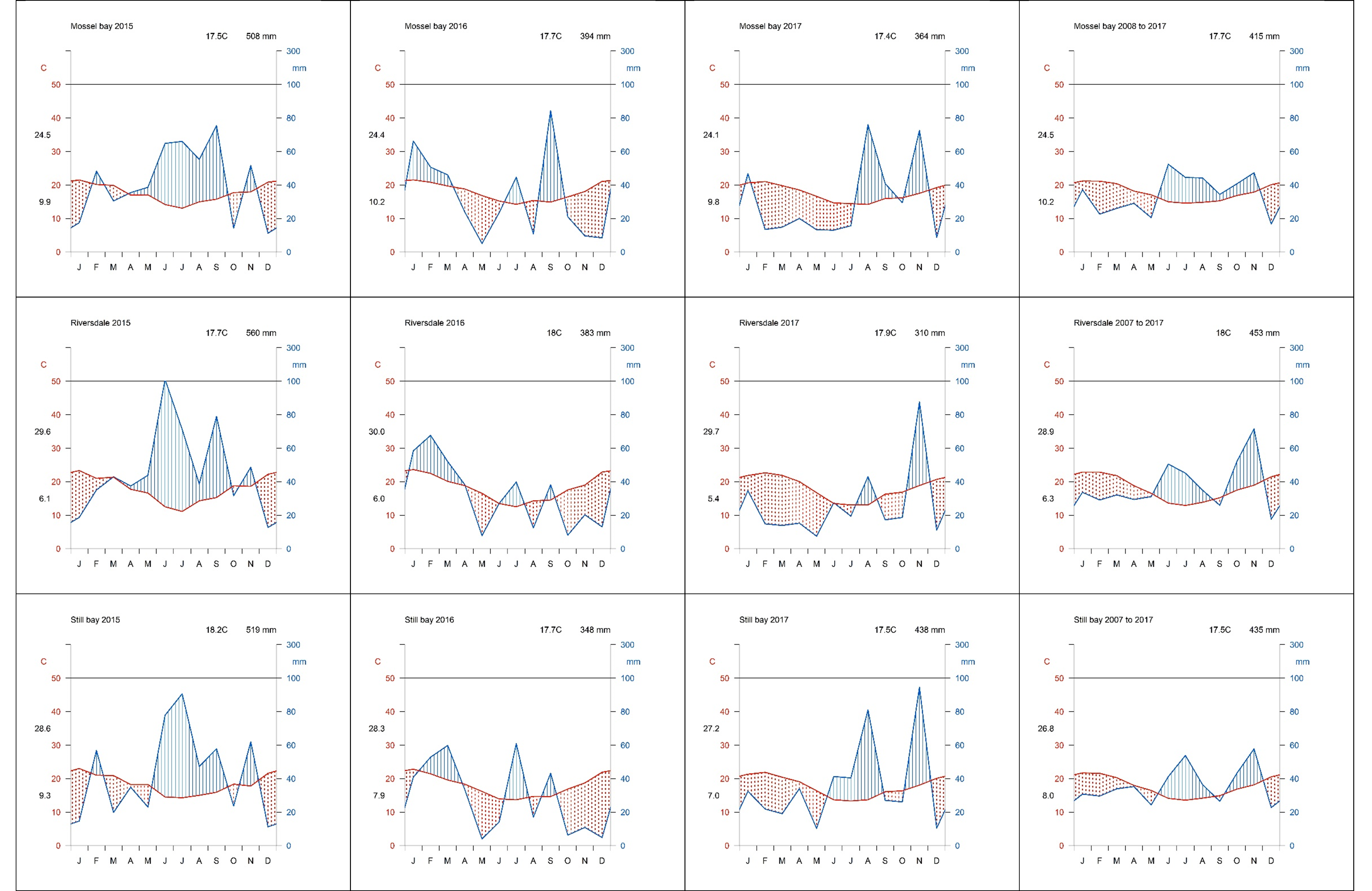

Supplement: Supplemental Information 3 — Mean temperature and rainfall indicated in right-hand corner of each plot. [file peerj-10-13066-s003.png]
