# Supplementary material for: The response of geophytes to continuous human foraging on the Cape south coast, South Africa and its implications for early hunter-gatherer mobility patterns
Source: PeerJ. 2022 May 3;10:e13066. doi: 10.7717/peerj.13066 (PMC9074880; doi:10.7717/peerj.13066)
Supplement: Supplementary file 4 [file peerj-10-13066-s004.docx]

**REASON**	**Plant/Forager Constraint**
Small plants do not have any food on them so it is not worth collecting them. "We are just hurting the plant."	Plant
They did not see it.	Forager
The extra effort to continue digging is not worth the return.	Plant
The foragers leave non-flowering and small plants behind.	Plant
Extra effort to break away dense, thorny bush clump to obtain edible food is not worth it.	Plant
Due to lower edible geophytes in the open parts of the plot, foragers put extra effort into breaking open bush clumps to reach geophytes.	Forager
They feel they have enough.	Forager
There is nothing left visible in the plot.	Plant
They drop some plants they had dug up, by accident.	Forager
Dried out vine of Cyphia digitata can not be traced down to the spot where it goes into the ground.	Plant
The whole plant or parts of it breaks off and are left behind when they dig for them.	Plant
They dig but the hard soil makes it impossible to extract the desired plant.	Plant
They are not as efficient as some other foragers.	Forager
Foragers not interested in these species (not well-known edibles).	Plant
Can not reach the site.	Plant
