# Supplementary material for: The response of geophytes to continuous human foraging on the Cape south coast, South Africa and its implications for early hunter-gatherer mobility patterns
Source: PeerJ. 2022 May 3;10:e13066. doi: 10.7717/peerj.13066 (PMC9074880; doi:10.7717/peerj.13066)
Supplement: Supplementary file 5 [file peerj-10-13066-s005.docx]

Species	Total number of plots in which the plant species occurred	Total weight harvested (g)	Percentage of total weight harvested (%)			Visual display of % weight harvested	Total number of plants harvested	Number of plants harvested per year			Proportion of plants not harvested by foragers			Reasons why the foragers did not harvest certain plants		
			2015	2016	2017			2015	2016	2017	2015	2016	2017	2015	2016	2017
**BULBS**																
Albuca fragrans	1	77.1	0	100	0		2	0	2	0		0				P
**SOFT TUBERS (SINGLE)**																
Cyphia digitata	5	2141	19.7	63.8	16.5		57	20	25	12	5	7	20	P, F	P	P
Kedrostis nana	2	1501.3	43.2	0	56.8		20	11	0	9	0		0	P	P	P
**SOFT TUBERS (SINGLE, MASS)**																
Satyrium carneum	1	3197.8	38.2	48.2	13.6		554	373	145	36	0	0	39	P	F	P
Satyrium longicolle	2	1018.4	51.4	42.8	5.8		382	243	128	11	21	54	95	P	P,F	P
**FIBROUS TUBERS (SINGLE)**																
Pelargonium lobatum	5	4518.2	41.1	49.7	9.2		191	89	66	36	0	6	0	P	P	P
Pelargonium rapaceum	3	338	81.2	18.8	0		64	62	2	0	0	0			F	P,F
Pelargonium triste	3	317.6	31.4	22.9	45.7		17	6	3	8	54	25	0	P	P, F	P
**CORMS (BUNCH)**																
Chasmanthe aethiopica	5	4676.4	65.7	30.6	3.7		868	701	136	31	4	23	11	P, F	P, F	P, F
Watsonia fourcadii	1	5474.9	69.7	29.5	0.8		357	294	56	7	0	0	0	P	F	P
Wachendorfia speciflora	1	24.9	100	0	0		41	41	0	0	0				P	
Watsonia sp	1	13.6	100	0	0		3	3	0	0	0				P	
**CORMS (SINGLE)**																
Babiana ambigua	3	233.6	8.7	23.3	68		55	5	6	44	0	14	43	P	P	F
Babiana patula	7	411.1	24.9	56.3	18.8		83	34	39	10	55	17	60	P	P, F	P, F
Babiana tubulosa	1	63.5	0	100	0		9	0	9	0		0				P
Ferraria crispa	1	843.7	88.7	0	11.3		324	296	0	28	0	100	3	P	F	P
Freesia alba	1	18.1	0	0	100		3	0	0	3	100		0	P	P, F	
Freesia sp	1	54.5	16.7	58.3	25		19	8	8	3	20	11	0	P	F	P
Gladiolus teretifolius	2	376.5	0	0	100		11	0	0	11			15	P		
Tritonia crocata	2	924.9	15.9	74.7	9.3		474	208	218	48	10	40	21	P	P, F	F
**ROOT**																
Conicosia pugioniformis	1	499	45.5	42.7	11.8		56	32	20	4	0	0	82	P	P	P
**LEAFY SPECIES**																
Mesembryanthemum atenus	1	2195.4	0	0	100		40	0	0	40			33	F		
Oxalis pes-caprae	1	2129.6	67.2	32.8	0		4793.6	798	3995.6	0	20	5	100	P	P	P
